# Prenatal Diagnosis and Outcome of Scimitar Syndrome: A Case Series of Six Patients

**DOI:** 10.3390/jcm11061696

**Published:** 2022-03-18

**Authors:** Florian Recker, Eva Christin Weber, Brigitte Strizek, Ulrike Herberg, Konrad Brockmaier, Ingo Gottschalk, Annegret Geipel, Ulrich Gembruch, Christoph Berg

**Affiliations:** 1 Department of Obstetrics and Prenatal Medicine, University of Bonn, 53127 Bonn, Germany; eva.weber@uk-koeln.de (E.C.W.); brigitte.strizek@ukbonn.de (B.S.); annegret.geipel@ukbonn.de (A.G.); ulrich.gembruch@ukbonn.de (U.G.); prof.berg@icloud.com (C.B.); 2 Division of Prenatal Medicine, Gynecological Ultrasound and Fetal Surgery, Department of Obstetrics and Gynecology, University of Cologne, 50937 Cologne, Germany; ingo.gottschalk@uk-koeln.de; 3 Department of Pediatric Cardiology, University of Bonn, 53127 Bonn, Germany; ulrike.herberg@ukbonn.de; 4 Department of Pediatric Cardiology, University of Cologne, 50937 Cologne, Germany; konrad.brockmeier@uk-koeln.de

**Keywords:** prenatal ultrasound, scimitar syndrome, partial anomalous pulmonary vein connection, congenital heart defect, fetal echocardiography

## Abstract

Scimitar syndrome is a rare disease characterized by hypoplasia of the right lung and partial anomalous pulmonary venous drainage to the inferior vena cava. All cases with a prenatal diagnosis of scimitar syndrome with or without associated malformations in an 18-year period (2000–2018) in two large tertiary referral centers (University of Bonn and University of Cologne, Germany) were retrospectively reviewed for the intrauterine course and postnatal outcome. Six cases were diagnosed in the study period. All presented with hypoplasia of the right lung, right-sided mediastinal shift, and abnormal pulmonary venous drainage to the inferior vena cava. Systemic arterial blood supply to the right lung, albeit postnatally present in all cases, could not be detected prenatally. Major associated anomalies were present in all cases and included atrial septal defect (*n* = 5), coarctation (*n* = 3), diaphragmatic hernia (*n* = 2), and VACTERL association (*n* = 1). Half of the cohort died within 6 months after birth and all three survivors suffer from long-term pulmonary sequelae. The primary hint to the prenatal diagnosis of scimitar syndrome is the abnormal position of the heart in the chest. If searched for, abnormal venous drainage can be identified prenatally and confirms the diagnosis. The prognosis depends on the presence of associated major anomalies and the need for neonatal intervention.

## 1. Introduction

Scimitar syndrome is an entity characterized by hypoplasia of the right lung and abnormal pulmonary venous drainage to the inferior vena cava with an incidence of 2:100,000 in live-born infants [1]. Abnormal arterial supply from the descending aorta to the hypoplastic lung is often present. Although Chasinat et al. [2] and Cooper et al. [3] reported the first cases in 1836, Neill et al. [4] described the scimitar sign and offered a detailed discussion of the syndrome. The term ‘scimitar’ represents the radiographic appearance of the anomalous venous drainage, likened to the silhouette of an oriental sword. Other associated malformations include bronchogenic cysts, horseshoe lung, accessory diaphragm, and hernias. In several patients with complete pulmonary vein transposition, the locus was mapped to the chromosome region 4q12 [5]. The clinical picture and the findings of transthoracic or transoesophageal echocardiography, angiography, computed tomography, and magnetic resonance angiography are used to make a postnatal diagnosis. Pryce [6] used the phrase “pulmonary sequestration” in 1946 to describe a “disconnected” bronchopulmonary mass with aberrant lung tissue without tracheobronchial connections and receiving systemic artery supply. Because of this latter characteristic, which encompasses both illnesses, scimitar syndrome patients are usually characterized postnatally as having a ‘sequestered lung’ [7]. Attempts at postnatal and prenatal classification of congenital anomalies of the bronchopulmonary tract and related vessels have been made, but the terminology remains confusing [7,8,9]. Prospective diagnosis of scimitar syndrome in the fetus has only been reported once [10], other accounts being isolated case reports diagnosed retrospectively [11,12,13,14]. There is one case by Michailidis et al. [15] where the diagnosis was just made retrospectively, aided by three-dimensional power Doppler imaging.

The more severe cases of scimitar syndrome present early in life, usually with dyspnea due to increased pulmonary blood flow secondary to the abnormal collateral artery supplying systemic arterial blood to the right atrium. Gao et al. reported the outcome of consecutive cases of scimitar syndrome presenting in infancy [16]. A large European multi-center registry study showed that respiratory (including respiratory distress, recurrent upper respiratory tract infections, cyanosis, and pneumonia) as well as cardiac symptoms (including failure to thrive and congestive heart failure) occur in about 50% of all clinical cases [17]. An Indian study focusing on surgical outcomes in scimitar patients even showed that associated cardiac and extracardiac defects, particularly hypoplasia of the right lung, are present in up to three-quarters of cases, whereas operative mortality has been cited between 4.8% and 5.9% [18]. The aim of this study was to assess the spectrum of extra-cardiac anomalies and chromosomal anomalies, the intrauterine course, and the postnatal outcome of fetuses with scimitar syndrome.

## 2. Materials and Methods

All cases with a prenatal diagnosis of scimitar syndrome with or without associated malformations in an 18-year period (2000–2018) in two tertiary referral centers for prenatal medicine (University of Bonn and University of Cologne, Germany) were retrospectively reviewed for intrauterine findings, course, and outcome. A prenatal diagnosis of scimitar syndrome was made in the presence of hypoplasia of the right lung resulting in ipsilateral mediastinal shifting and abnormal pulmonary venous drainage to the inferior vena cava. All cases underwent a complete fetal anatomic survey that included fetal echocardiography and Doppler sonography in a standardized fashion. Fetal echocardiography was carried out by a segmental approach using standardized anatomical planes incorporating pulsed-wave and color Doppler imaging (15). For all ultrasound examinations, 5-, 7.5-, or 9-MHz curved array probes were used (IU22, Philips, Hamburg, Germany; Voluson E8 and E10, GE Healthcare, Solingen, Germany). A postmortem or postpartum examination to verify the ultrasound results was performed in all cases. One case from the series has already been published as a case report [19].

As the Ethics Committees of the University of Bonn and Cologne do not request formal approval for an anonymized retrospective analysis of clinical data, ethical consent was not sought.

## 3. Results

**Figure 1:**
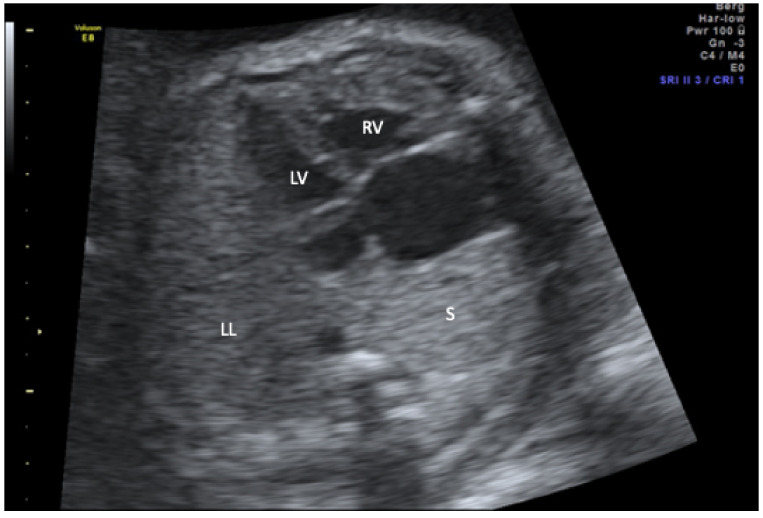
Transverse thoracic view in b-mode demonstrating dextroposition of the heart and a small echogenic mass that represents the sequestration (S) behind the heart on the right side. LL, left lung; LV, left ventricle; RV, right ventricle.

**Figure 2:**
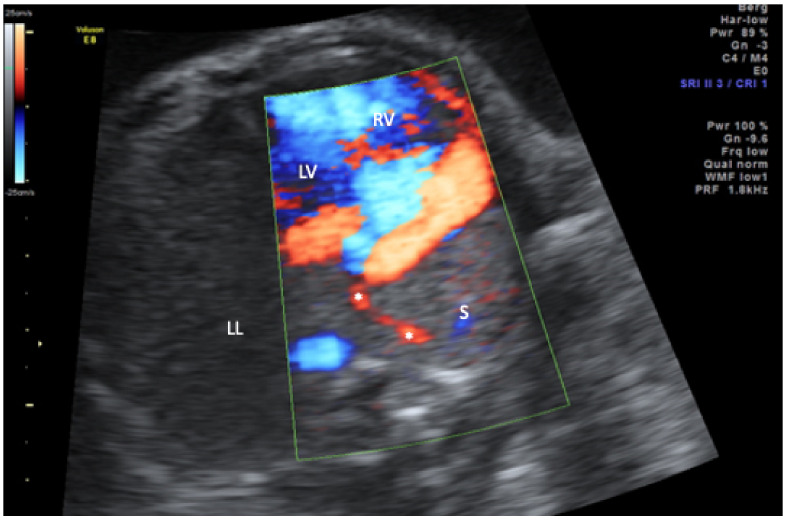
Transverse thoracic view in color-mode demonstrating the scimitar vein (asterisk) draining the sequestration (S) into the inferior vena cava (not displayed). LL, left lung; LV, left ventricle; RV, right ventricle.

**Figure 3:**
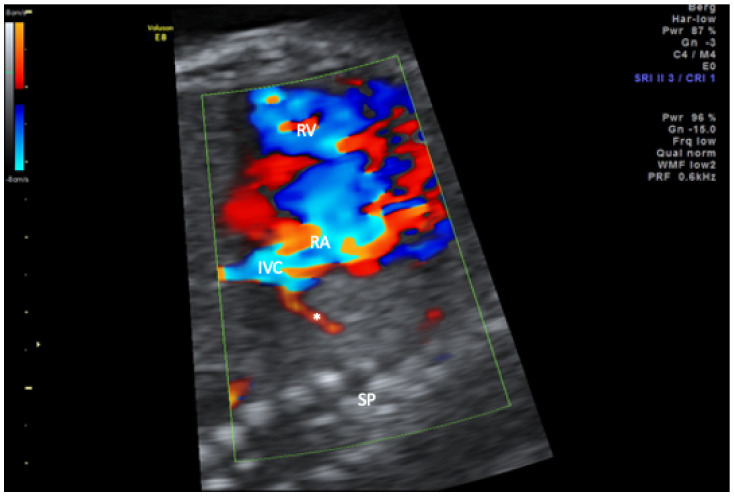
Sagittal view of the thorax in color-mode demonstrating the scimitar vein (asterisk) draining into the inferior vena cava (IVC) in close proximity of the right atrium. RA, right atrium; RV, right ventricle; SP, spine.

In the study period, six affected singleton pregnancies were diagnosed in our centers. The average maternal age at diagnosis was 30 years. All couples were healthy and nonconsanguineous. The median diagnosis was made at 26.3 (range 17.2–38.0) weeks of gestation. Three of the diagnoses were made upon second-trimester screening and the remaining three in the third trimester. In the third trimester cases, a complete organ screening including fetal Doppler sonography and fetal echocardiography was also performed as in the second-trimester screening. Five of the six fetuses presented with a combination of mediastinal shift, hypoplasia of the right lung, and right-sided partially anomalous pulmonary venous drainage to the vena cava inferior (Figure 1, Figure 2 and Figure 3).

Only in one case with VACTERL association and diaphragmatic hernia was the scimitar vein missed at prenatal scans, although mediastinal shift and hypoplasia of the right lung were present and the drainage of the right pulmonary veins could not be ascertained. In this case, the prenatal diagnosis was finally left in doubt. A systemic abnormal blood supply to the right lung, albeit postnatally present in all of our cases, could not be demonstrated only in one case at prenatal scans. In one case, a retrospective video analysis showed an arterial supply from coeliac trunk to the lower part of the left lung with typical pulmonary high resistance blood flow pattern. Additional prenatally detected major anomalies included coarctation in three cases and diaphragmatic hernia in two cases, one of the latter in the fetus with VACTERL association, anal atresia, hemivertebrae, and renal dysplasia. In one case with diaphragmatic hernia and coarctation, a duplication of 10q22.1q23.2 was detected, which has been reported to be frequently associated with cardiac defects [20]. In all cases, a right-sided pulmonary sequestrum with supplying systemic arteries and anomalous venous drainage was found postnatally. The arterial supply originated either from the truncus celiacus or directly from the abdominal aorta or from the thoracic aorta in two cases each. A secundum atrial septal defect was postnatally diagnosed in five of the six cases. All pregnancies ended with live births and in all cases where a catheter-guided occlusion of the systemic supply to the sequestrum was performed in the neonatal period. Half of the cases were fatal within the first six months. One infant died after only one month with pulmonary and intracranial hemorrhage and suspected brainstem damage. Another died due to intractable pulmonary hypertension at the age of six months. The remaining case died after six months following reinsertion of the left pulmonary veins and reconstruction of the aortic arch with postoperative hydrocephalus due to ventilation malfunction.

Among the three survivors, one is living with severe limitations, including infantile cerebral palsy and with the help of a tracheostoma and a PEG device. Another is suffering from recurrent bronchial stenosis requiring multiple bronchial stent placements. The remaining survivor is thriving with only mild pulmonary hypertension (Table 1).

## 4. Discussion

The most important pointer for the prenatal diagnosis of scimitar syndrome is an abnormal position of the heart in the thorax with right-sided mediastinal shift. In these cases, a thorough search for abnormal pulmonary venous drainage is warranted in order to establish the diagnosis and to distinguish other causes of dextroposition. The detection of abnormal arterial pulmonary supply has proven difficult if not impossible in our current as well as previously published series and is therefore an important discriminator from bronchopulmonary sequestration where the arterial supply can be mapped in most cases [10]. In scimitar syndrome, the failure to visualize the arterial supply may be due to hypoplasia of this part of the lung and high vascular resistance in fetal life, such that the extent of blood supply to the abnormal lung is insufficient to allow prenatal visualization by color Doppler echocardiography, just as in the five cases described by Bhide et al. [10]. A right-sided mediastinal shift (ipsilateral to the affected lung) and normal pulmonary echogenicity may be more suggestive of scimitar syndrome, whereas pulmonary sequestration is more likely to have a contralateral mediastinal shift and focal increase in pulmonary echogenicity.

Even if the visualization of the scimitar vein fails, scimitar syndrome should be considered in the presence of right mediastinal shift with an intact diaphragm and a narrow right pulmonary artery [14]. Other sonographic signs may include: absence of a connection between the pulmonary veins and the left atrium, venous confluence posterior to the left atrium, a smooth posterior wall of the left atrium, abnormal Doppler waveforms, and dilated superior caval or brachiocephalic veins [21]. If a diagnosis of scimitar syndrome is considered based on prenatal investigations, an early postnatal examination should be performed in order to concretize the vascular anatomy and, if necessary, to perform an interventional cardiac catheterization [22].

Scimitar syndrome is often associated with additional anomalies, which include secundum atrial septal defect, diaphragmatic hernia, tetralogy of Fallot, and coarctation. As corroborated in our series, scimitar syndrome may belong to the cardiac malformations of the VACTERL association [19]. In general, surgical correction is recommended in all symptomatic patients and asymptomatic patients with pulmonary-to-systemic flow ratios greater than 1.5:1 or pulmonary-to-systemic flow ratios lesser than 1.5:1 in the settings of clinically treated pulmonary hypertension, stenosis of the scimitar vein, or concomitant cardiac lesions [18,23].

Scimitar syndrome must be differentiated from pseudoscimitar syndrome, in which an abnormal descending vein pursues an aberrant course in the right lung but drains normally into the left atrium, and from Kartagener’s syndrome [21]. Unilateral agenesis of the right lung also results in a variable ipsilateral mediastinal shifting; however, in these cases, the supplying and draining vessels are not visible and ipsilateral extrapulmonary anomalies are frequent [22].

Postnatally, plate atelectasis should also be considered. In this case, there is usually an atypical course of the pulmonary vein, usually horizontal or laterally ascending, with regular venous drainage into the left atrium, as well as an isolated pulmonary sequestrum, usually fed from the aorta [24].

The outcome of prenatally diagnosed scimitar syndrome has previously been reported to be quite favorable. Paladini et al. summarized four prenatal series including thirteen cases, of which ten (76.9%) survived [25]. However, six of these cases did not warrant being operated on and associated anomalies were rare, therefore representing the mild end of the spectrum. The outcome in our cohort was significantly worse; however, all fetuses had associated major anomalies and a neonatal intervention was warranted in every case. Therefore, our cohort rather represents the severe end of the spectrum. Due to the screening modalities, there is no specific visualization of the pulmonary veins. Therefore, only cases with a pronounced mediastinal shift to the right are usually detected prenatally. This may be the reason why, at least in the present study, the prenatal diagnosis of scimitar syndrome with a pronounced mediastinal shift had an unfavorable outcome. This is, in contrast to the course of cases of scimitar syndrome, diagnosed in childhood or late in life. This is also the reason why the diagnosis of left-sided scimitar syndrome is usually not made prenatally. In these cases, there is at most a minimal mediastinal shift, which is usually not recognized due to the correct position of the heart axis.

In summary, the diagnosis of scimitar syndrome should be considered in cases with otherwise unexplained right-sided mediastinal shift with hypoplasia of the right lung and the corresponding pulmonary artery. Detection of right-sided anomalous pulmonary venous drainage confirms the diagnosis. The prognosis depends on the presence of associated major anomalies and the need for neonatal intervention.

## Figures and Tables

**Table 1:** Presentation of the prenatal and postnatal characteristics of fetuses with scimitar syndrome.

Case	GA Diagnosis	Prenatal Findings	Delivery	Additional Postnatal Findings	Follow Up
1	34 + 1	mediastinal shift; dextroposition of the heart, right pulmonary hypoplasia, partial anomalous pulmonary drainage (scimitar vein)	39 + 1	Feeding vessel from coeliac trunk, secundum atrial septal defect	Coil occlusion of feeding vessel, pulmonary hemorrhage with respiratory deterioration, intracranial hemorrhage, diabetes insipidus centralis, exitus at 1 month
2	38 + 0	mediastinal shift; dextroposition of the heart, right pulmonary hypoplasia, partial, anomalous pulmonary drainage (scimitar vein), right sided diaphragmatic hernia; coarctation; duplication 10q22.1–10q23.2	39 + 2	Feeding vessel from abdominal aorta, pulmonary hypertension	Coil occlusion of feeding vessel, intractable pulmonary hypertension, exitus at 6 months
3	20 + 2	mediastinal shift; dextroposition of the heart, right pulmonary hypoplasia, partial anomalous pulmonary drainage (scimitar vein)	35 + 5	Feeding vessel from abdominal aorta, hypoplastic aortic arch; secundum atrial septal defect; anomalous supracardiac pulmonary drainage of left pulmonary veins in brachiocephalic vein	Plug occlusion of feeding vessel, reinsertion of left pulmonary veins, patch reconstruction of aortic arch, postoperative hydrocephalus; hypoplastic corpus callosum; ventilation malfunction hypoxic crisis with bradyasystole, exitus at 6 months,
4	31 + 1	mediastinal shift; dextroposition of the heart, right pulmonary hypoplasia, anal atresia, diaphragmatic hernia, hemivertebrae, single umbilical artery, renal dysplasia	33 + 6	partial anomalous pulmonary drainage (scimitar vein), Feeding vessels from thoracic aorta, hypoplastic aortic arch, secundum atrial septal defect, VACTERL association	Plug occlusion of feeding vessel and 2 MAPCAs in neonatal period, multiple bronchial stent placements, 4 years old
5	22 + 2	mediastinal shift; dextroposition of the heart, right pulmonary hypoplasia, partial anomalous pulmonary drainage (scimitar vein)	31 + 0	Feeding vessel from coeliac trunk, secundum atrial septal defect, mild coarctation	Plug occlusion of feeding vessels in neonatal period, thriving with mild pulmonary hypertension, 7 years old
6	17 + 2	mediastinal shift; dextroposition of the heart, right pulmonary hypoplasia, partial anomalous pulmonary drainage (scimitar vein), single umbilical artery, left persistent superior caval vein	40 + 0	2 Feeding vessels from thoracic aorta, anomalous drainage of right sided pulmonary veins in left atrium, secundum atrial septal defect	Plug occlusion of feeding vessels in neonatal period and at age of one year. Correction of right pulmonary veinous drainage with intra-atrial tunnel and ligation of persisting ductus arteriosus at age of 2 years, infantile cerebral palsy; tracheostoma; Percutaneous endoscopic gastrostomy(PEG) placement, 10 years old

## Data Availability

Not applicable.
